# Psychometric properties of Parenting Sense of Competence Scale using item-response theory

**DOI:** 10.1016/j.heliyon.2024.e38212

**Published:** 2024-09-24

**Authors:** Mitra Rahimzadeh, Sara Esmaelzade Saeieh, Parisa Rezanejad-Asl

**Affiliations:** a Social Determinants of Health Research Center, Department of Biostatistics and Epidemiology, School of Health, Alborz University of Medical Sciences, Karaj, Iran; b Department of Midwifery, School of Medicine, Social Determinants of Health Research Center, Alborz University of Medical Sciences, Karaj, Iran; c Non-Communicable Diseases Research Center, Department of Biostatistics and Epidemiology, School of Health, Alborz University of Medical Sciences, Karaj, Iran

**Keywords:** Psychometrics, Parental competence, Item response theory

## Abstract

Parental competence is one of the essential components of raising ethical and healthy children. The mother's satisfaction with her maternal role significantly influences her cognitive responses and parenting behaviors. Having appropriate instruments to measure maternal satisfaction and identify and solve potential problems after childbirth can substantially contribute to maintaining and improving mother and infant health. This study aimed to evaluate the psychometric properties of the Parenting Sense of Competence (PSOC) Scale in mothers referring to the healthcare centers affiliated with Qazvin University of Medical Sciences. This cross-sectional psychometric study collected data from 254 mothers with 1.5-month-old infants referred to the healthcare centers in Qazvin Province. Data were analyzed using the item response theory (IRT) in SPSS 26 and Stata 17 software. The mean age (±standard deviation) of participants was 30.05 ± 5.7, and the overall Cronbach's alpha for the scale was 0.931. We used the graded response model (GRM) appropriate for Likert-type rating scales to fit the IRT. The discrimination parameter estimates showed that item 5 had a discrimination level <0.65, leading to its exclusion from the final analysis. In addition, the total information index confirmed that the scale was suitable in the trait range of −2.1 to 1.8. Compared with other common models for ordered responses based on general model fit indices, the GRM showed a better fit. The study results recommend applying the IRT models to improve and enhance the quality of questionnaires in various measurement fields.

## 1 Introduction

Using questionnaires and surveys to measure cognitive characteristics such as knowledge, attitude, performance, and competence is very common in studies related to health, medicine, psychology, and other fields. Measuring these concepts is challenging because they have multidimensional and multifaceted natures. Moreover, latent variables cannot be measured directly; instead, they can be indirectly measured through multiple observed variables called items and the scores obtained from these items, which are ultimately considered indicators of unobserved constructs.

Spearman introduced classical test theory (CTT) in the context of scores obtained from tests [1]. Despite the longstanding use of CTT in developing, administering, and analyzing tests, it has some limitations. For more details, please refer to the following articles [[2], [3], [4], [5]].

Despite the limitations, Hambleton and Jones highlight its usability and easy result analysis compared to other theories as significant advantages of CTT, making it more widely utilized than other theories [6]. Moreover, CTT has had an important role in developing other theories, including item response theory (IRT). The relationship between latent variables and observed scores can be nonlinear in IRT. Additionally, the estimation of true scores is independent of the sample used, allowing for straightforward generalization of results.

In 1960, a Danish mathematician, George Rasch, introduced IRT to analyze responses to a series of reading tests [7]. At the University of Chicago, Wright developed Rasch's advanced models and analytical tools and enhanced their application across various scientific domains [8]. The IRT aims to address the limitations of CTT models significantly with the following assumptions.

1. There is an item characteristic curve (ICC) between the measured latent trait and the probability of responding to a specific item in a particular category [9,10].
2. The unidimensionality of the measured trait implies a common factor justifying all covariances between items; that is, a common factor explains all the covariances in the items, which is the latent trait [6]. However, one can employ the following multidimensional models if this assumption is unmet [11].
3. The local independence assumption suggests that responses to test items are statistically independent; only the examinee's ability level is the primary factor. If this assumption exists, the examinee's performance must not be influenced by their right or wrong answers to the questions [12].

While Rasch's model was initially designed for dichotomous responses, various models have been developed for polytomous responses, including the graded response model (GRM) (Samejima, 1969), nominal response model (NRM) (Bock, 1972), rating scale model (RSM) (Andrich, 1978a), partial credit model (PCM) (Masters, 1982), and generalized partial credit model (GPCM) (Muraki, 1992) [[13], [14], [15], [16], [17]].

The IRT encompasses a set of mathematical models that describe the relationship between an individual's "ability" or "trait" and their responses to questions on a measurement scale. This relationship is depicted through an ICC. Theoretically, this curve, which is monotonically increasing, illustrates the relationship between the probability of a correct response to a question (right-wrong) and various ability levels. As ability increases, the likelihood of a correct response increases. The theoretical range of ability is considered to be between -∞ and +∞, although it ranges from −3 to 3 in practice [18].

When questionnaire items have more than two response options, the interpretation of ICCs slightly varies as they depict the expected score within the range of the targeted trait [19]. Key parameters in the IRT model include item discrimination (a), item difficulty or location (b), guessing (c), and trait score (θ). The discrimination parameter, determining the slope of the curve, indicates the question's power to discriminate examinees at different trait levels. Larger values make the question's characteristic curve closer to the vertical axis, while smaller values make it closer to the horizontal axis. The difficulty parameter represents the trait level at which the probability of a correct response is 50 % for these examinees, provided the guessing parameter is zero. The guessing parameter indicates the likelihood of a correct response to an item at the lowest ability level and is not applicable in models with Likert-type scales [20].

In the two-parameter model in IRT, the probability of correct answer to each item is obtained as follows:

$$\mathrm{Pr}\left(Y_{ij}=1|\theta_{j}\right)=\frac{\exp\left\{a_{i}\left(\theta_{j}-b_{i}\right)\right\}}{1+\exp\left\{a_{i}\left(\theta_{j}-b_{i}\right)\right\}}\;\theta_{j}∼N\left(0,1\right)$$

Where $a_{i}$ is a discrimination parameter for r, $b_{i}$ is the difficulty parameter, and $\theta_{j}$ is the trait parameter of the jth individual to choose the correct answer. If we add the guess parameter to this model, the probability of correct answer for each item will be obtained as follows:

$$\mathrm{Pr}\left(Y_{ij}=1|\theta_{j}\right)=c_{i}+\left(1-c_{i}\right)\frac{\exp\left\{a_{i}\left(\theta_{j}-b_{i}\right)\right\}}{1+\exp\left\{a_{i}\left(\theta_{j}-b_{i}\right)\right\}}\;\theta_{j}∼N\left(0,1\right)$$

In this model, $c_{i}$ is the guess parameter. This model, presented by Birnbaum (1968), has used the logistic link function, which is a generalization of the model presented by Lord (1952) who used the normal link function [21,22].

The GRM model is a family of polynomial models in item-response theory (IRT) and is useful when multiple answers to an item are ordinal, such as the Likert scale. This model is a direct extension of the above two-parameter model and is based on the hypothesis that the discrimination parameter in different items is unequal. This parameter shows the severity of the relationship between an item and the measured construct or latent trait. In addition, this model assumes that the distance between response categories is not the same in all cases.

In this model, each item has k ordinal responses, and a discrimination parameter is estimated for each item. It also includes k-1 difficulty parameters at the threshold points used to separate boundaries between ordinal responses. The probability that the k^th^ or higher answer for the i^th^ item is chosen by the j^th^ person is obtained from the following formula:

$$\mathrm{Pr}\left(Y_{ij}\geq k|\theta_{j}\right)=\frac{\exp\left\{a_{i}\left(\theta_{j}-b_{ik}\right)\right\}}{1+\exp\left\{a_{i}\left(\theta_{j}-b_{ik}\right)\right\}}\;\theta_{j}∼N\left(0,1\right)$$

Where $a_{i}$ is a discrimination parameter for r, $\theta_{j}$ is the trait parameter of the j^th^, and $b_{ik}$ is discrimination parameter at the threshold points used to separate boundaries between ranked responses. The following formula obtains the probability that the k^th^ or higher answer for the i^th^ item is chosen by the j^th^ person:

$$\mathrm{Pr}\left(Y_{ij}=k|\theta_{j}\right)=\mathrm{Pr}\left(Y_{ij}\geq k|\theta_{j}\right)-\mathrm{Pr}\left(Y_{ij}\geq k+1|\theta_{j}\right)$$

$$\mathrm{Pr}\left(Y_{ij}=1|\theta_{j}\right)=\frac{\exp\left\{a_{i}\left(\theta_{j}-b_{i}\right)\right\}}{1+\exp\left\{a_{i}\left(\theta_{j}-b_{i}\right)\right\}}\;\theta_{j}∼N\left(0,1\right)$$

$\mathrm{Pr}\left(Y_{ij}\geq 0\right)=1$ and $\mathrm{Pr}\left(Y_{ij}>k\right)=0$ mean that the probability of a person choosing the first or greater answer is one and the probability of choosing an answer greater than K will be zero. If the responses are bimodal, this model is reduced to 2 parameters presented by Birnbaum (1968) [21].

The item information function indicates the accuracy of each question and the entire questionnaire in detecting and estimating the desired trait; larger values represent more information. This index is typically reported along with its standard error, with an inverse relationship [19].

The IRT models have been increasingly employed in education, psychology, health, and biostatistics [[23], [24], [25], [26], [27]]. Becoming a mother results in fundamental changes in cognitive, socio-emotional, and behavioral functions. A would-be mother must add the motherhood identity to her existing identities, establish a deep emotional connection with her child, and be capable of meeting the child's needs [28]. Parental competence is a mother's belief in her ability to perform maternal tasks accurately. Additionally, maternal satisfaction refers to the mother's sense of calmness and contentment in her role as a parent [29]. In all societies, motherhood is considered the most significant anticipated role for many women, although some may not choose it or may delay adopting it [30,31]. However, it is more often inevitable [32]. Motherhood is an essential transition for a woman, adding new responsibilities to her life [29].

The motherhood role is influenced by various factors, including a woman's age, economic status, educational background, social and cultural factors, perception of childbirth experience, and psychological status and characteristics after becoming pregnant. Among several predictive factors related to maternal role adaptation, a woman's identity as a mother is crucial. A woman with a positive maternal identity is generally more competent in caring for her infant. When a mother aligns with her role, she can confidently utilize her capabilities to foster the infant's physical, emotional, behavioral, and social growth [29]. Considering that playing the role of a mother in the early years of the child requires spending a lot of energy and time, the satisfaction of taking on this task based on the mother's emotional and spiritual needs can increase the mothers' sense of competence and worthiness [33].

The increased competence of mothers, especially primigravida, significantly impacts the psychosocial development of infants. Social support can also be a crucial factor in becoming a mother. Acquiring competence and satisfaction from the maternal role is essential to acquiring the mother role. The birth of an infant is not only a significant event for the parents but also a concern for the entire family [34].

Based on the motivation theory proposed by White, the capacity of a person to do the work (competence) and his satisfaction with the way of doing the work (satisfaction) are the two main components of doing a good job. One type of motivation is competency motivation, which is called efficiency and performance by White [35].

Mark believes that the competency of the mother's role reflects her belief in her ability and satisfaction to effectively perform the mother's role, which refers to her feeling of the child care with comfort and satisfaction. Competence in the maternal role develops when mothers have the knowledge and skills to care for their children [36]. The results of studies have shown that most mothers acquire the necessary skills to take care of their infants during postpartum [37]. However, some mothers need more support to accept the motherhood role. The lack of support from the family and the health service system negatively impacts family relationships and the relationship between mother and infant. For this reason, it is necessary to identify mothers who cannot adapt themselves to the role of motherhood in the early-postpartum [38,39]. A systematic review showed that early identification, meeting the needs, culture, family support, and postnatal midwifery homecare were essential factors in the transition to motherhood [40].

The Parental Sense of Competence Scale (PSOC) by Gibaud-Wallston is a 17-item scale measuring parenting efficacy and satisfaction subscales. Each item is rated on a 6-point Likert scale, from "strongly disagree" to "strongly agree." The total scale score ranges from 17 to 102, with a higher score indicating greater competence. Items #2, 3, 4, 5, 8, 9, 12, 14, and 16 are reversely coded [41]. This questionnaire was further validated in a subsequent study by Johnston and Mash in 1989 [42], and item 17 was removed, resulting in a 16-item scale. In a 2015 study in Iran, Kordi et al. assessed its reliability using Cronbach's alpha, yielding a value of 0.75 [43]. Moreover, another Iranian study on women in the postpartum period has approved the reliability of this scale [44].

Different countries have studied the reliability and validity of this scale. For example, a study conducted on Chinese women in a 1-month and 3-month postpartum found that the reliability values ranged from 0.72 to 0.89 for the efficacy domain and 0.84 to 0.85 for the satisfaction domain [45]. In addition, another study in China conducted a study on mothers recruited at the postnatal unit and presented Cronbach’s alpha of 0.85 for the scale and 0.8 and 0.77 for the efficacy and satisfaction subscales [46]. A study in the United States conducted on women at 10 weeks of postpartum revealed that the reliability indices of efficacy, satisfaction, and total scale were 0.83, 0.80, and 0.87 [47]. Finally, a study on the parents of children aged 3–6 years found Cronbach's alpha values of 0.89 and 0.9 for mothers and 0.89 and 0.87 for fathers regarding satisfaction and efficacy [48]. Considering several studies using the 16-item scale, this study employed this version and assessed its psychometrics using the IRT [36,[49], [50], [51]].

The aim of this study was to assess the psychometric properties of the Parental Sense of Competence Scale using IRT.

## 2 Materials and methods

The sampling was conducted in multiple stages. First, we chose 5 out of 12 centers in Qazvin city in the west, east, south, north, and center. In the second stage, sampling was done through convenience sampling, and the mothers of 1.5-month-old infants referring for vaccination completed the self-reporting questionnaire. We selected 60 mothers from each center and obtained informed written consent from them. Finally, they completed the demographic information and PSOC questionnaires.

The inclusion criteria were the absence of specific diseases in mothers with healthy and full-term infants. On the other hand, the exclusion criteria embraced those who had high-risk pregnancies, postpartum depression, a history of psychiatric medication use, and hospitalization of the mother or infant in ICUs.

Data were analyzed in SPSS 26 and STATA 17 using factor analysis and IRT. This study is part of a master's thesis in Midwifery Counseling, approved by the Ethics Committee of Alborz University of Medical Sciences with the ethics code IR.ABZUMS.REC.1399.259.

## 3 Results

The data from 254 mothers with 1.5-month-old infants referred to the healthcare centers in Qazvin city were collected from 300 distributed questionnaires, with an approximate response rate of 85 %. The participating mothers had a mean age of 30.05 ± 5.7 years (17–46 years), 84.3 % were housewives, 52.4 % had university degrees, 36.6 % had unplanned pregnancies, 36.6 % experienced vaginal childbirth, 78.3 had no pregnancy complication, 90.9 % received help for childcare besides the spouse, 8.3 % had their husband's help only, 0.8 % mentioned that they had no help for childcare, 17.7 % of the newborn infants needed to be hospitalized, 50.4 % had only this infant, and 91.3 % breastfed. According to the calculations, 53 % were male infants, and the mean ± standard deviation of birth weight was 3.06 ± 0.56 kgs (1.05–4.28 kgs), with 43.3 % weighing between 3 and 3.5 (Table 1).

**Table 1:** Demographic characteristics of the mothers and new borne in study.

Variables	Frequency	Percent
**Education M**		
Under diploma	48	18.9
Diploma	75	29.5
University	133	52.4
**Job M**		
Housewife	214	84.3
Others		15.7
**Baby Gender**		
Boy	135	53.1
Girl	119	46.9
**Baby Weight**		
<2.5 kg	40	15.7
2.5–3	57	22.5
2.5 - 3.5	110	43.3
>3.5	47	18.5
**Breastfeeding**		
Yes	232	91.3
No	22	8.7
**Delivery Type**		
Natural	93	36.6
**Cesarean section**	161	63.4
**NICU Hospitalization**		
Yes	45	17.7
No	209	82.3
**Pregnancy Type**		
Wanted	161	63.4
Unwanted	93	63.4
**Number of Children**		
One	128	50.4
Two	84	33.1
Three and more	42	16.5
**Having help for childcare**		
Just spouse	21	8.3
Spouse and others	231	90.9
No	2	0.8

Table 2 presents each item's mean (SD) and item-total correlation, showing the highest mean value for the first and the lowest for the eighth items.

**Table 2:** Descriptive indices of parental sense of competence scale items with item-scale correlation and the alpha if item deleted.

Item #	mean	SD∗	Item–total correlation	α if item deleted
Item 1	**4.799**	1.662	0.618	0.927
Item 2	4.327	1.873	0.793	0.922
Item 3	3.252	1.744	0.600	0.928
Item 4	4.402	1.902	0.790	0.922
Item 5	3.000	1.613	−0.007	0.941
Item 6	3.713	1.740	0.804	0.922
Item 7	3.535	1.819	0.753	0.924
Item 8	**2.906**	1.629	0.324	0.934
Item 9	3.362	1.768	0.648	0.926
Item 10	3.827	1.713	0.771	0.923
Item 11	4.134	1.646	0.636	0.927
Item 12	4.500	1.705	0.632	0.927
Item 13	3.823	1.808	0.759	0.923
Item 14	4.130	1.928	0.666	0.926
Item 15	3.909	1.739	0.815	0.922
Item 16	4.614	1.749	0.781	0.923

### 3.1 Standard deviation

The questionnaire's reliability, measured by Cronbach's alpha, was 0.931, and the most significant change occurred by removing item 5, increasing it to 0.941 (Table 2).

**Fig. 1:**
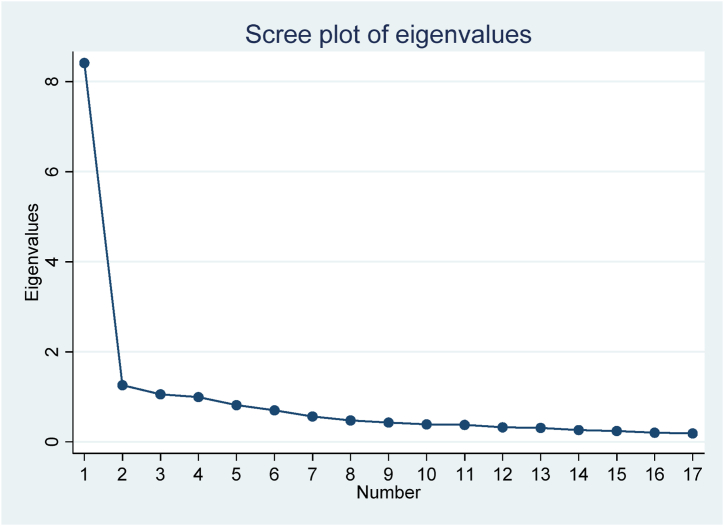
Scree plot.

Before fitting the IRT model, initial assumptions of the model were examined. The unidimensionality of the PSOC was investigated using exploratory factor analysis. The obtained results from eigenvalues (Table 3) and the scree plot (Fig. 1) indicate the unidimensionality of the intended factor. According to Reckase (2006), the assumption of unidimensionality is met if the first factor explains at least 20 % of the total test variance or the eigenvalue of the first factor is at least three times that of the subsequent factor [11].

**Table 3:** Eigenvalues and explained variance percentages in exploratory factor analysis.

Component	Eigen Value	Of %Variance
1	8.4	52.47
2	1.25	7.82
3	1.04	6.47

Table 3 reports the estimated values for factor eigenvalues. This table shows that the value of the first factor's eigenvalue is 8.4, explaining more than 50 % of the total variance.

Hamilton states that if the assumption of unidimensionality holds, the assumption of local independence will also be valid [12]. Extracting a dominant factor reduces the covariance between variables to zero or near zero, and its removal eliminates the relationship between items. Given sufficient evidence for unidimensionality, we will accept the local independence assumption.

Table 4 demonstrates the common variance of each questionnaire item. Items with a common variance of less than 0.5 are removed in exploratory factor analysis [52]. As this table shows, none of the items have a common variance of less than 0.5; item 5 has the highest common variance.

**Table 4:** Common variance of each item in principal component analysis.

Item #	Communality extraction
Item 1	0.506
Item 2	0.715
Item 3	0.582
Item 4	0.704
Item 5	0.780
Item 6	0.758
Item 7	0.654
Item 8	0.623
Item 9	0.621
Item 10	0.704
Item 11	0.624
Item 12	0.608
Item 13	0.714
Item 14	0.644
Item 15	0.768
Item 16	0.677

Table 5, Table 6 report the discrimination coefficients, threshold coefficients, and information values calculated for the PSOC Scale. Table 5 shows the discrimination coefficients in descending order. The lowest is 0.055 for item 5, while the highest is 3.48 for item 15. The discrimination coefficients of other items fall between these two values. Baker and Kim state that discrimination parameters <0.65 are considered low, between 0.65 and 1.34 are moderate, and ≥1.35 are high [53]. Based on this criterion, item 5 (My mother was better prepared to be a good mother than I am), with a discrimination coefficient <0.65, is excluded from the questionnaire items. Moreover, item 8 (A difficult problem in being a parent is not knowing whether you’re doing a good job or a bad one.) has a moderate discrimination index, yet it is retained in the scale because its significance is < 0.001.

**Table 5:** Estimation of discrimination parameter (standard deviation) with Wald statistics and significance level.

Item #	Discrimination	Std	Wald statistics	Sig
Item 5	0.055	0.127	0.44	0.66
Item 8	0.810	0.143	5.67	<0.001
Item 12	1.635	0.192	8.5	<0.001
Item 3	1.638	0.182	8.99	<0.001
Item 1	1.654	0.196	8.43	<0.001
Item 14	1.745	0.197	8.85	<0.001
Item 9	1.839	0.200	9.19	<0.001
Item 11	2.053	0.216	9.52	<0.001
Item 16	2.586	0.279	9.26	<0.001
Item 7	2.777	0.292	9.50	<0.001
Item 2	2.969	0.313	9.48	<0.001
Item 10	3.058	0.311	9.83	<0.001
Item 4	3.148	0.343	9.19	<0.001
Item 13	3.319	0.341	9.73	<0.001
Item 6	3.401	0.344	9.88	<0.001
Item 15	3.496	0.359	9.72	<0.001

**Table 6:** Estimation of difficulty parameters in the GRM Model (without item 5).

Item #	b1(≥2)	b2(≥3)	b3(≥4)	b4(≥6)	b5(6)
Item 8	−1.960	−1.414	1.587	1.844	2.338
Item 12	−2.449	−1.100	−0.652	−0.520	0.326
Item 3	−1.448	−0.272	0.587	0.717	1.195
Item 1	−1.803	−1.431	−1.369	−0.741	0.100
Item 14	−1.676	−0.787	−0.362	−0.259	0.366
Item 9	−1.575	−0.371	0.497	0.645	0.908
Item 11	−1.502	−0.956	−0.764	−0.032	1.105
Item 16	−1.882	−1.198	−0.565	−0.387	0.036
Item 7	−0.714	−0.447	−0.283	0.445	1.091
Item 2	−1.592	−0.743	−0.287	−0.212	0.208
Item 10	−0.980	−0.593	−0.446	0.244	1.127
Item 4	−1.425	−0.758	−0.288	−0.195	0.104
Item 13	−0.917	−0.435	−0.328	0.151	0.954
Item 6	−0.939	−0.493	−0.284	0.330	1.107
Item 15	−0.997	−0.589	−0.466	0.107	1.057

The values related to the difficulty parameter were estimated with five parameters for each item based on the 6-point Likert scale. These values indicate the probability of selecting the options, and their suitability is unrelated to their quantity. The most suitable cases for these coefficients are their symmetry and the normality of their distribution. Items 3, 6, 9, 13, and 15 in the scale demonstrate threshold coefficients of symmetry.

**Fig. 2:**
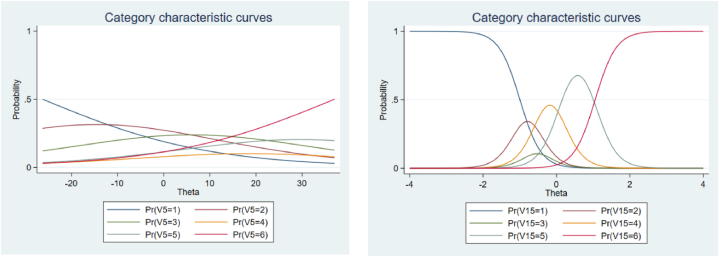
The left panel shows item characteristic curve for item 5 (worst items) and the right panel shows item characteristic curve for item 15 (best items).

Fig. 2 depicts the item characteristic curve for items 5 and 15. As observed in this figure, as the value of the discrimination parameter increases (item 15), the category-response curve becomes more compressed, and its height increases. Conversely, when the value of the discrimination parameter is smaller, the curve appears parallel to the horizontal axis.

**Fig. 3:**
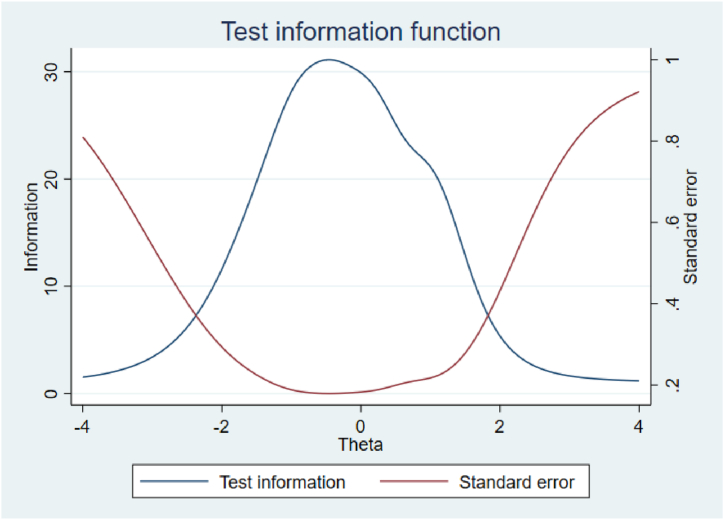
The information curve of the entire scale.

Fig. 3 presents all items' effectiveness information and the scale's overall information function and standard deviation. The highest is achieved at θ = −0.25; overall, the scale effectively distinguishes individuals whose current trait is between −2.1 and 1.8.

Considering that PSOC is a six-point Likert scale, we fitted four existing models, including the GRM, RSM, PCM, and GPCM, for rank-ordered responses to the data. Item 5 had low discrimination coefficients, so it was eliminated in the analysis of all four models. We used fitting indices, such as the Akaike information criterion (AIC) and Bayesian information criterion (BIC), to compare different models. Based on these indices, the best model has the lowest fitting index value [13]. According to Table 7, the GRM model has a better fit than other models.

**Table 7:** Fit indices for the overall model across five different models.

Model	ll(model)	df	AIC	BIC
GRM	−4832.54	90	**9845.08**	**10163.44**
GRM(a = 2.4)	−4933.972	75	10017.94	10283.24
PCM	−4990.07	76	10132.14	10400.98
GPCM	−4898.504	90	9977.008	10295.37
RSM	−5425.912	20	10891.82	10962.57

To examine a model with a consistent discrimination level for all items, we fitted a model to the data with a consistent discrimination level and the mean discrimination of all items (except item 5). Although this model has 15 fewer parameters than the model with different discrimination coefficients, it has a higher fitting index. Moreover, the likelihood ratio test indicates that the model with different discrimination coefficients significantly fits better at the 0.0001 significance level.

## 4 Discussion and conclusion

This study aimed to evaluate the psychometric properties of the PSOC scale using the IRT model in mothers with 1.5-month-old infants. It sought to eliminate inappropriate items and obtain a questionnaire with fewer items and higher accuracy. The best model (GRM) results were analyzed based on the remaining 15 items, with discrimination coefficients exceeding the acceptable minimum of 0.65. All items, except item 8, had discrimination coefficients >1.35, indicating the power of these questions in discriminating the individuals with different latent traits. Item 8, with a discrimination parameter of 0.81, had the average value, and we decided to keep it on the scale apart from its statistical significance and consistency with the literature review. Correspondingly, the concept of this item had an important message about the mother's critical view toward her ability to care for her child.

In addition, the total information fit (TIF) plot showed that the PSOC performs well for individuals with latent traits (θ) in the range of −2.1 to 1.8, with the highest information obtained at θ = −0.25. Considering that the maximum range for the latent trait is −3 to 3 in practice, the scale used for assessing parental competence in individuals with various latent trait values demonstrates suitable and acceptable performance.

Several studies have used explanatory or confirmatory factor analyses. It is worth mentioning that these methods, which are a branch of CTT, consider the relationship between the factors and components linearly, while the logistic link function is used in the IRT models because they are more flexible than the linear model. To the best of our knowledge, no study has assessed the psychometric properties of this scale with the IRT models. Most studies, assuming one hidden trait, have confirmed a 16-item scale with two components, which is different from the IRT model we used in this study [[54], [55], [56], [57]].

Several studies have removed some items and obtained a new version of scale; they have some similarities and differences. Similar results have been obtained in a study on Australian parents with 6-month to 15-year-old children. In this study, item 5 was deleted from the set of items both in mothers and fathers, but item 8 was deleted in the mothers and remained in the fathers. It is worth mentioning that they used the 17-item scale, but item 17 was removed in both groups at the beginning of the analysis [51]. In addition, they used the explanatory factor analysis (without items 5 and 8) and found that the three components could account for 51.6 % variances. However, our study revealed one component with an eigenvalue of more than one, accounting for 59.16 % of variances without items 5 and 8. These results present sufficient evidence that a unique latent trait can explain the competence in our study.

A study on Spanish mothers with 6- to 12-month-old children used the 17-item scale. They employed confirmatory factor analysis, removed item 17, and extracted two components. Correspondingly, item 8 had a factor loading of less than 0.3, but the authors decided to keep all items in the scale [56]. Another study on Spanish mothers removed items # 2, 5, 7,8, 12, 14, and 17 and confirmed a 10-item scale with two components. Exploratory factor analysis showed that these two components could explain less than 50 % of variances [58].

Two studies worked on the Portuguese version of this scale. The first study with 146 at-risk mothers used the 16-item scale. Its results differed from our study (Nunes et al., 2016), and they removed two items (# 8 and16). The second study (Nunes et al., 2022), which assessed PSOC among a Portuguese sample of community and at-psychological-risk parents, removed 6 items (# 1, 2, 6, 8, 12, and 16) from the scale and confirmed two subscales with 5 items in each [59,60].

The difference between the two groups led to the change in the psychometric results of the scale, which is the main weakness in the CTT; the validity and reliability depend on the sample [2]. Delhalle et al. came up with results that differed from those of our studies. They removed 3 items (# 2,6, 7) because of cross-loading (>0.20) [61]. Gilmore et al. (2009) removed items 1, 5, and 7 in a factor analysis with 3 components. Likewise, our study removed item 5; however, items 1 and 7 remained [62]. In addition, Gilmore et al. involved a reevaluation of the psychometrics of this scale. They devised a new version considering that all positive items were loaded on one subscale and all negative items on another. This revised questionnaire consists of 16 items, with 10 items related to the efficacy domain and six to the satisfaction domain. Psychometric indices, including Cronbach's alpha, were calculated, resulting in 0.91 and 0.88 for efficacy and satisfaction domains and 0.92 for the overall scale. Test-retest results over 3–5 months yielded scores of 0.75 and 0.81 for efficacy and satisfaction domains and 0.79 for the overall scale [63]. Since this scale is new, its reliability and validity in the different languages and cultures should be evaluated carefully and extensively.

IRT models have been extensively employed in psychology and are expanding in healthcare domains. However, their application is limited because they do not exist in SPSS and Prism. Moreover, these models demand a deeper understanding of theoretical and mathematical concepts than classical test models, contributing to their lesser adoption in the medical and healthcare fields. Notably, these models enable more precise measurements while reducing respondent burden by identifying and excluding less informative or relevant items, resulting in shorter questionnaires.

Using this theory in the healthcare and medical fields requires special attention. In the classical test method, Cronbach's alpha is used to examine the consistency of questionnaire items. Therefore, each item is eliminated individually, and Cronbach's alpha is calculated for the remaining items. If there is a significant increase in the alpha value, omitting that item can improve the questionnaire [64]. The present study conducted a sensitivity analysis, revealing the most significant change for item 5. Although removing this item increased Cronbach's alpha by 0.016, it was not removed since the change was negligible. However, the discrimination coefficients for this item were very low in the IRT model, leading to their removal from the scale.

The limitations of this study included its small sample size, the investigation of the mothers with 1.5-month-old infants, and the use of convenience sampling. It is recommended that we use heterogeneous samples with a sample size of at least 300 in IRT models [65]. Despite these limitations, the evidence of the validity of the scale indicates that the model fits the data well.

## Data availability statement

The data supporting the findings of this study are available from the corresponding author upon reasonable request.

## CRediT authorship contribution statement

**Mitra Rahimzadeh:** Writing – original draft, Supervision. **Sara Esmaelzade Saeieh:** Validation, Investigation, Data curation. **Parisa Rezanejad-Asl:** Writing – review & editing, Methodology, Formal analysis.

## Declaration of competing interest

The authors declare that they have no known competing financial interests or personal relationships that could have appeared to influence the work reported in this paper.

## References

[1] Lord F.M., Novick M.R. (2008).

[2] De Champlain A.F. (2010). A primer on classical test theory and item response theory for assessments in medical education. Medical education.

[3] Hu Z. (2021). The integration of classical testing theory and item response theory. Psychology.

[4] Idaka I., Idaka E. (2014). From classical test theory (CTT) to item response theory (IRT) in research instrument. Lwati A J. Contemp. Res..

[5] Smiley J. (2015). Classical test theory or Rasch: a personal account from a novice user. Shiken.

[6] Hambleton R.K., Jones R.W. (1993). Comparison of classical test theory and item response theory and their applications to test development. Educ. Meas..

[7] Rasch G. (1966). An item analysis which takes individual differences into account. Br. J. Math. Stat. Psychol..

[8] Massof R.W. (2002). The measurement of vision disability. Optom. Vis. Sci..

[9] Lord F.M. (2012).

[10] Embretson S.E., Reise S.P. (2000).

[11] Reckase M.D. (2006). Multidimensional item response theory. Handb. Stat..

[12] Hambleton R.K., Swaminathan H. (2013).

[13] Andrich D. (1978). Application of a psychometric rating model to ordered categories which are scored with successive integers. Appl. Psychol. Meas..

[14] Darrell Bock R. (1972). Estimating item parameters and latent ability when responses are scored in two or more nominal categories. Psychometrika.

[15] Masters G.N. (1982). A Rasch model for partial credit scoring. Psychometrika.

[16] Muraki E. (1992). A generalized partial credit model: application of an EM algorithm. Appl. Psychol. Meas..

[17] Samejima F. (2016).

[18] Baker F.B. (2001).

[19] Raykov T., Marcoulides G.A. (2018).

[20] Osteen P. (2010). An introduction to using multidimensional item response theory to assess latent factor structures. J. Soc. Soc. Work. Res..

[21] Birnbaum A. (1968). Some latent trait models and their use in inferring an examinee's ability. Statistical theories of mental test scores.

[22] Lord F.M. (1952).

[23] Ayanwale M.A., Isaac-Oloniyo F.O., Abayomi F.R. (2020). Dimensionality assessment of binary response test items: a non-parametric approach of bayesian item response theory measurement. Int. J. Eval. Res. Educ..

[24] Chiarotto A. (2018). Item response theory evaluation of the biomedical scale of the Pain Attitudes and Beliefs Scale. PLoS One.

[25] Jeong H.-J. (2020). An application of item response theory to scoring patient safety culture survey data. Int. J. Environ. Res. Publ. Health.

[26] Tutz G. (2021). Hierarchical models for the analysis of Likert scales in regression and item response analysis. Int. Stat. Rev..

[27] Zanon C. (2016). An application of item response theory to psychological test development. Psicol. Reflexão Crítica.

[28] Dashevsky A. (2012).

[29] Shrestha S. (2019). Maternal role: a concept analysis. Journal of Midwifery & Reproductive Health.

[30] Jahanbakhshi A. (2023). Childbearing tendency and related factors among married women in rasht city, north of Iran. Journal of Holistic Nursing And Midwifery.

[31] Szekeres H., Halperin E., Saguy T. (2023). The mother of violations: motherhood as the primary expectation of women. Br. J. Soc. Psychol..

[32] Vieira M.E.B., Linhares M.B.M. (2011). Developmental outcomes and quality of life in children born preterm at preschool-and school-age. J. Pediatr..

[33] Mortazavi F. (2014). Maternal quality of life during the transition to motherhood. Iran. Red Crescent Med. J..

[34] Saeieh S.E. (2017). Perceived social support and maternal competence in primipara women during pregnancy and after childbirth. International journal of community based nursing and midwifery.

[35] White R.W. (1959). Motivation reconsidered: the concept of competence. Psychol. Rev..

[36] Márk-Ribiczey N., Miklósi M., Szabó M. (2016). Maternal self-efficacy and role satisfaction: the mediating effect of cognitive emotion regulation. J. Child Fam. Stud..

[37] Hudson D.B., Elek S.M., Fleck M.O. (2001). FIRST-TIME MOTHERS'AND FATHERS'TRANSITION to parenthood: infant care self-efficacy, parenting satisfaction, and infant sex. Issues Compr. Pediatr. Nurs..

[38] Forster D.A. (2008). The early postnatal period: exploring women's views, expectations and experiences of care using focus groups in Victoria, Australia. BMC Pregnancy Childbirth.

[39] Hamzehgardeshi Z. (2018). Adaptation to motherhood and its influential factors in the first year postpartum in Iranian primiparous. Preventive Care in Nursing & Midwifery Journal.

[40] Walker S.B., Rossi D.M., Sander T.M. (2019). Women's successful transition to motherhood during the early postnatal period: a qualitative systematic review of postnatal and midwifery home care literature. Midwifery.

[41] Gibaud-Wallston J., Wandersmann L.P. (1978).

[42] Johnston C., Mash E.J. (1989). A measure of parenting satisfaction and efficacy. J. Clin. Child Psychol..

[43] Fasanghari M., Kordi M., Asgharipour N. (2019). Effect of maternal role training program based on Mercer theory on maternal self-confidence of primiparous women with unplanned pregnancy. J. Educ. Health Promot..

[44] Bagherinia M., Meedya S., Mirghafourvand M. (2018). Association between maternal sense of competence and self-efficacy in primiparous women during postpartum period.

[45] Ngai F.-W., Chan S.W.-C., Holroyd E. (2007). Translation and validation of a Chinese version of the parenting sense of competence scale in Chinese mothers. Nursing research.

[46] Zhu Y. (2022). Parenting sense of competence and its predictors among primiparous women: a longitudinal study in China. BMC Pregnancy Childbirth.

[47] Copeland D.B., Harbaugh B.L. (2004). Transition of maternal competency of married and single mothers in early parenthood. J. Perinat. Educ..

[48] Matalon C., Turliuc M.N. (2023). Parental self-efficacy and satisfaction with parenting as mediators of the association between children's noncompliance and marital satisfaction. Curr. Psychol..

[49] Bui E. (2017). Psychometric properties of the parenting sense of competence scale in treatment-seeking post-9/11 veterans. J. Child Fam. Stud..

[50] Kristensen K.B. (2023). Parents with a mental illness and their sense of parenting competence. Advances in Mental Health.

[51] Rogers H., Matthews J. (2004). The parenting sense of competence scale: investigation of the factor structure, reliability, and validity for an Australian sample. Aust. Psychol..

[52] Gebremedhin M., Gebrewahd E., Stafford L.K. (2022). Validity and reliability study of clinician attitude towards rural health extension program in Ethiopia: exploratory and confirmatory factor analysis. BMC Health Serv. Res..

[53] Baker F.B., Kim S.-H. (2004).

[54] Curci S.G., Luecken L.J., Edwards M.C. (2021). Psychometric properties of the parenting sense of competence scale among low-income mexican american mothers. J. Child Fam. Stud..

[55] Ohan J.L., Leung D.W., Johnston C. (2000). The Parenting Sense of Competence scale: evidence of a stable factor structure and validity. Canadian Journal of Behavioural Science/Revue canadienne des sciences du comportement.

[56] Oltra‐Benavent P. (2020). Spanish version of the Parenting Sense of Competence scale: evidence of reliability and validity. Child Fam. Soc. Work.

[57] Suwansujarid T. (2013). Validation of the parenting sense of competence scale in fathers: Thai version. Southeast Asian J. Trop. Med. Publ. Health.

[58] Menendez S., Jimenez L., Victoria Hidalgo M. (2011). Factorial structure of PSOC (Parental Sense of Competence) scale with a sample of mothers from family preservation services. REVISTA IBEROAMERICANA DE DIAGNOSTICO Y EVALUACION-E AVALIACAO PSICOLOGICA.

[59] Nunes C. (2022). Healthcare.

[60] Nunes C. (2016). Psychometric properties of an adapted version of the parental sense of competence (PSOC) scale for P ortuguese at‐risk parents. Child Fam. Soc. Work.

[61] Delhalle M. (2024). Psychometric properties of the parenting sense of competence scale for a sample of French-speaking mothers. J. Child Fam. Stud..

[62] Gilmore L., Cuskelly M. (2009). Factor structure of the parenting sense of competence scale using a normative sample. Child: care, health and development.

[63] Gilmore L., Cuskelly M. (2023). The parenting sense of competence scale: updating a classic. Child Care Health Dev..

[64] Hajjar S. (2018). Statistical analysis: internal-consistency reliability and construct validity. International Journal of Quantitative and Qualitative Research Methods.

[65] Dai S. (2021). Performance of polytomous IRT models with rating scale data: an investigation over sample size, instrument length, and missing data. Front Educ.

