# Supplementary material for: Psychometric properties of Parenting Sense of Competence Scale using item-response theory
Source: Heliyon. 2024 Sep 24;10(19):e38212. doi: 10.1016/j.heliyon.2024.e38212 (PMC11466605; doi:10.1016/j.heliyon.2024.e38212)
Supplement: Multimedia component 1 [file mmc1.docx]

Parenting Sense of Competence Scale with 16 items

(Gibaud-Wallston & Wandersman, 1978)

Strongly Disagree (1) Somewhat Disagree (2) Disagree (3)

Agree (4) Somewhat Agree (5) Strongly Agree (6)

**1.** The problems of taking care of a child are easy to solve once you know. how your actions affect your child, an understanding I have acquired

**2.** Even though being a parent could be rewarding, I am frustrated now. while my child is at his / her present age.

**3.** I go to bed the same way I wake up in the morning, feeling I have not. accomplished a whole lot.

**4.** I do not know why it is, but sometimes when I’m supposed to be in control, I feel more like the one being manipulated.

**5**. **My mother was better prepared to be a good mother than I am.**

**6.** I would make a fine model for a new mother to follow in order to 6. learn what she would need to know in order to be a good parent.

**7.** Being a parent is manageable, and any problems are easily solved.

**8. A difficult problem in being a parent is not knowing whether you’re doing a good job or a bad one.**

**9.** Sometimes I feel like I’m not getting anything done.

**10.** I meet by own personal expectations for expertise in caring for my child.

**11.** If anyone can find the answer to what is troubling my child, I am the one.

**12.** My talents and interests are in other areas, not being a parent.

**13.** Considering how long I’ve been a mother, I feel thoroughly familiar

with this role.

**14.** If being a mother of a child were only more interesting, I would be motivated to do a better job as a parent.

**15.** I honestly believe I have all the skills necessary to be a good mother to my child.

**16.** Being a parent makes me tense and anxious.

**17.** Being a good mother is a reward in itself. **(removed item in 16 item scales)**
